# Long-term treatment with subcutaneous immunoglobulin in multifocal motor neuropathy

**DOI:** 10.1038/s41598-021-88711-9

**Published:** 2021-04-28

**Authors:** Luca Gentile, Massimo Russo, Carmelo Rodolico, Ilenia Arimatea, Giuseppe Vita, Antonio Toscano, Anna Mazzeo

**Affiliations:** 1 Unit of Neurology and Neuromuscular Diseases, Department of Clinical and Experimental Medicine, University of Messina, Messina, Italy; 2 UOC Neurologia e Malattie Neuromuscolari, Policlinico G. Martino, via Consolare Valeria 1, 98124 Messina, ME Italy

**Keywords:** Neuroscience, Peripheral nervous system

## Abstract

Multifocal motor neuropathy (MMN) is a rare disease with a prevalence of less than 1 per 100,000 people. Intravenous immunoglobulin (IVIG) therapy, performed for a long-term period, has been demonstrated able to improve the clinical picture of MMN patients, ameliorating motor symptoms and/or preventing disease progression. Treatment with subcutaneous immunoglobulin (SCIg) has been shown to be as effective as IVIG. However, previously published data showed that follow-up of MMN patients in treatment with SCIg lasted no more than 56 months. We report herein the results of a long-term SCIg treatment follow up (up to 96 months) in a group of 8 MMN patients (6 M; 2F), previously stabilized with IVIG therapy. Clinical follow-up included the administration of Medical Research Council (MRC) sum-score, the Overall Neuropathy Limitation Scale (ONLS) and the Life Quality Index questionnaire (LQI) at baseline and then every 6 months. Once converted to SCIg, patients’ responsiveness was quite good. Strength and motor functions remained stable or even improved during this long-term follow-up with benefits on walking capability, resistance to physical efforts and ability in hand fine movements.

## Introduction

Multifocal motor neuropathy (MMN) is a rare disorder, affecting peripheral motor nerves, with a prevalence ranging from 0.29 to 0.70 per 100,000^1^. MMN is more frequent in men than in women (ratio of 2.7:1)^2^ and 80% of patients experienced their first symptoms at a relatively young age (20–50 years)^3^. The most common presenting symptom is asymmetric distal weakness (wrist drop, reduced grip strength, foot drop) due to impairment of single nerve’s function^3,4^. A retrospective study of 88 MMN patients demonstrated the presence of symptoms at lower limbs in up to one-third of cases^1^. Minor sensory symptoms have been observed in at least 20% of patients^3^.

The diagnosis of MMN is based on the clinical and electrodiagnostic criteria developed by the European Federation of Neurological Societies (EFNS) and the Peripheral Nerve Society (PNS) Task Force in 2006 and revised in 2010^1^. The hallmark of MMN is the most frequent neurophysiological sign, i.e., the multifocal presence of persistent, partial conduction blocks (CB) of motor axons, without signs of demyelination. For definition, CB should be searched outside the usual sites of nerve compression^5^. The 2010 guidelines also reported four supportive criteria: (1) elevated IgM antiganglioside GM1 antibodies; (2) increased cerebrospinal fluid (CSF) protein (< 1 g/l); (3) MRI showing increased signal intensity on T2-weighted imaging; (4) objective clinical improvement following intravenous immunoglobulin therapy (IVIG) treatment^6^.

The course of MMN is often slowly progressive. However, a patient could also present a step-wise progression. MMN diagnosis can be a challenge, especially outside referral centers for neuromuscular disorders, with a mean delay of 4 years from symptoms onset to diagnosis^5^. The natural history of MMN is unfavourable, with most of the patients gathering progressive disabilities that highly compromise their daily-life activities. However, some patients have a favourable long-term prognosis in absence of specific therapy or may have occasionally a spontaneous remission^3^.

Various immunomodulatory treatments have been used in MMN patients. Some of them (glucocorticoids, plasma exchange, rituximab, cyclophosphamide and mycophenolate mofetil) have showed poor or any efficacy at all, when they have not even worsened the course of disease (plasma exchange and corticosteroids). Moreover, seeing as their potentially dangerous side effects, their use is not recommended^4^. IVIG, performed for long-term period, has been demonstrated able to improve the clinical picture of MMN patients, ameliorating motor symptoms and/or preventing disease progression. IVIG are usually administered at a dose of 2 g/kg perfused over a 4–5 days period. IVIG cycles are periodically repeated, with a frequency that depends on personal responsiveness to the treatment. Commonly, most of the patients need an IVIG cycle every 4–6 weeks, and this implies that patients must frequently be admitted to the hospital. This, altogether with venous access problem or possible systemic adverse reactions to IVIG infusion, have been reported as the main reason of complain to the treatment^7^. In patients with MMN, treatment with subcutaneous immunoglobulin (SCIg) has been shown to be as effective as IVIG in improving motor parameters or in stabilizing patients’ clinical condition. In open-labelled studies, a long-term follow-up for one to two years has shown that SCIg can maintain muscle strength and prevent relapse of symptoms^8^. In addition, SCIg delivery, after an adequate training of the patient, can be independently performed at home, without the necessity of any venous access^7^. It has to be outlined that, during IVIG courses, variations of hemoglobin levels and of other hemolytic parameters have been detected. About this side effect, it has been observed a better tolerability after having switched from IVIG to more frequent but smaller SCIg injections^9^. The majority of patients reached clinical stability using a SCIg dose equivalent to IVIG, although this aspect seems to be in contrast with previous reports suggesting the need of an increase of SCIg dosage^10^. However, it appears from the literature data that the longest SCIg treatment follow-up lasted no longer than 56 months^11^. We report herein the retrospective results of a long-term SCIg treatment in a group of MMN patients, with a follow up period up to 8 years, including safety, tolerability, clinical outcome measures variations and patients’ perception of SCIg treatment.

## Patients

We retrospectively examined eight patients, all > 18 year-old, with a diagnosis of MMN defined according to the EFNS/PNS criteria. They were successfully treated with IVIG (2 g/kg in 4–5 days) with stabilization of clinical conditions. All patients started IVIG administration every 4/6 weeks before switching to SCIg treatment. SCIg infusions were performed with an interval (2–3 times/week) that differed from patient to patient, depending on the intervals between previously administered IVIG courses and on the quantity of immunoglobulin perfused. A SCIg dosage equivalent to IVIG (1:1) was used.

## Methods

Study procedures were the same of a previously published study on chronic inflammatory demyelinating polyradiculoneuropathy (CIDP) patients^12^. First SCIg injection was administered at the hospital under surveillance of a study nurse. Subsequently, injections were controlled at home via a programmable infusion pump (crono-speed 50 by Canè S.p.A, Italy) coupled with a 50 ml syringe connected with catheters to a butterfly subcutaneous needle. The nurse was always available for contacts if needed^12^.

All patients signed an informed consent form and the study has been approved by the Ethics Committee of the University Hospital of Messina (address: AOU “G. Martino”, via Consolare Valeria n. 1, 98125-Messina (ME), Italy. E-mail: aoucomitatoetico@unime.it). This protocol has been performed in accordance with the ethical standards laid down in the 1964 Declaration of Helsinki and its later amendments. We considered baseline records and follow-up data collected between 2012 and 2020. During this period, patients were evaluated at baseline and every 6-months by the same neurologist. They were also interviewed by the study nurse as regard as quality of life and on feasibility, safety and side effects at baseline and at the following visits^12^.

Clinical follow-up included:

1. Medical Research Council (MRC) sum-score (MRC-SS) to check muscle strength (0 = complete paralysis, 80 = normal strength) bilaterally in eight muscle groups (shoulder abduction, elbow flexion, wrist extension, index finger abduction, hip flexion, knee extension, foot dorsiflexion and great toe dorsiflexion)^12^.
2. The Overall Neuropathy Limitation Scale (ONLS: 0 = normal, 11 = worst) to assess motor disability^12^.
3. The Life Quality Index questionnaire (LQ I) as a quality of life measures. 15-items examining the respondents perception of immunoglobulins treatment impact on daily activities, summarized to four sub-scales: “treatment interference”, “therapy related problems”, “therapy setting” and “treatment costs”^12^.

Patients with ONLS reduction of at least 1 point were considered improved. Neither MRC score nor ONLS scale variations were considered as evidence of strength or motor ability stabilization. In case of adverse reactions, deterioration of muscle strength or development of paresthesias, the patients were revaluated. Relapses were identified as clinical deterioration with increase of ONLS and decrease of MRC-SS at least of one point. In case of relapse, SCIg dose was increased by 25% for 4 weeks and the patient was then reassessed. If the increase did not produce clinical improvement, an IVIG course was administered to the patient, not interrupting SCIg treatment. The patient was then revaluated after 2 weeks: if the clinical condition was improved, IVIG course was not repeated and only SCIg treatment was continued, at pre-relapse dose^12^.

## Results

### Demographic and disease history

At baseline, the eight patients (6 M; 2 F) had a mean age of 55.9 years (years) (range 31–80 years) and they all presented with a long history of MMN (mean age at disease onset: 44.6 years) (Table 1). Two patients were also affected by diabetes type 2, and one of the two also had a cutaneous T-cell lymphoma, whereas a third one was affected by an autoimmune hypothyroidism. Five patients correctly received the diagnosis of MMN at first, and they began a specific treatment a mean of 1.5 years after onset (range 0–2.5 years). On the other hand, three patients were misdiagnosed as having a cervical radiculopathy, a right plexopathy/motor neuron disease or a carpal tunnel syndrome. These mistakes led to a considerable delay of MMN diagnosis and treatment (mean delay: 8.5 years; range 5–11 years).

**Table 1:** Clinical and demographic characteristics of MMN patients.

Pt	Age at baseline (years)	Sex	Comorbidity	Age at disease onset	First diagnosis	Onset symptoms	Secondary symptoms	Sensory symptoms	Latency onset/secondary symptoms	Latency onset/FLT	Present phenotype
1	53	F	–	36 yrs	Carpal tunnel syndrome	Fasciculations UL and LL	Proximal right UL weakness and burning	Yes	3 yrs	11 yrs	Proximal and distal right and left UL weakness, distal left LL weakness
2	74 (d)	M	Diabetes, cutaneous t cell lymphoma	64 yrs	MMN	Distal right UL weakness and paresthesias	Distal left UL weakness and paresthesias	Yes	1 yrs	0	Tetraparesis
3	48	F	–	40 yrs	Cervical radiculopathy	Distal right UL weakness	Proximal right UL weakness ad cramps	–	5 yrs	5 yrs	Weakness Proximally at right UL and lower limbs and distally at upper limbs and left LL
4	80 (d)	M	Autoimmune hypothyroidism	58 yrs	MMN	Fasciculations, proximal left UL weakness, distal left LL weakness, dysesthesias at left hand and foot	Distal right UL weakness and paresthesias	Yes	5 yrs	2.5 yrs	Proximal weakness at left UL, distal weakness at right UL and lower limbs
5	59	M	–	52 yrs	Right plexopathy/2nd motoneuron disease	Distal right UL weakness	–	–	–	9.5 yrs	Distal right UL weakness
6	61	M	Diabetes	45 yrs	MMN	Proximal right UL weakness and pain	Proximal left UL weakness and pain, motor disturbances at lower limbs	–	1 month	2 yrs	Proximal and distal weakness at the 4 limbs
7	31	M	–	29 yrs	MMN	Distal left LL weakness	Distal left UL weakness	–	1 yrs	1 yrs	Distal weakness at left UL and lower limbs
8	41	M	–	33 yrs	MMN	Proximal right LL weakness	Proximal right and left UL weakness, distal right UL weakness	Yes	7 yrs	2 yrs	Right hemiatrophy of the tongue. Proximal and distal upper and lower limbs weakness. Hypoesthesia in right ulnar nerve territory and from both knees to feet

*Pt* patient, *(d)* dead, – not applicable. *Yrs* years, *MMN* multifocal motor neuropathy, *UL* upper limb, *LL* lower limb, *FLT* first line treatment.

The onset of symptoms was characterized by distal weakness in 5/8, proximal weakness in 2/8 and fasciculations in 1/8. After the disease onset, all patients but one complained of secondary symptoms as new motor deficits or sensory disturbances, with a mean latency of 2.75 years (1 month–7 years) (Table 1). At onset, or even as secondary symptoms, three patients complained of pain or cramps at proximal limbs. Only the patient #8 showed sensory deficits (hypoesthesia and hypopallesthesia at right ulnar nerve and at lower limbs from the knee to feet). At the last neurological examination before starting treatment, in comparison with neurological evaluation at onset, 7/8 patients showed a progression of the disease. On the other hand, patient #5 maintained the same neurological features since onset.

### First/second/third line treatments

IVIG were used as first line treatment in 5/8, always with good response (Table 2). On the other hand, patient #2, #4 and #6, before starting IVIG, had been treated with cyclophosphamide 300 mg/die respectively for 6 years, 6 months and 12 years globally. However, these periods are not to be intended as consecutive: once disease regression was reached, cyclophosphamide was stopped and restarted only in case of relapses. This treatment was then definitely interrupted because of side effects (hemorrhagic cystitis in two cases, leukopenia and thrombocytopenia in the other). These three patients, after cyclophosphamide, underwent other immunomodulatory treatment (rituximab, azathioprine, interferon-beta) with no or only partial benefits. IVIG treatment was then started for all three patients. Globally, the mean duration of IVIG treatment in this group of eight patients was of 6.5 years (range 1–19 years).

**Table 2:** Type and length of treatment courses.

Pt	First line treatment (FLT)	FLT duration (years)	FLT efficacy	Second and third line treatment	Second and third treatment efficacy	IVIG duration (years)
1	IVIG	6	Yes (stabilization)	–	–	6
2	Ciclophosfamide	6	Yes (complete regression)	IVIG	Yes	4
3	IVIG	3	Yes (initial almost complete regression)	–	–	3
4	Ciclophosfamide	0.5	Yes (almost complete regression)	(1) AZT, (2) interferon beta, (3) IVIG	Partial/no/yes	19
5	IVIG	7	Yes (initial complete regression, then partial benefit)	–	–	7
6	Ciclophosfamide	12	Yes (almost complete regression)	(1) Rituximab, (2) IVIG	Partial/yes	2
7	IVIG	1	Yes (initial almost complete regression)	–	–	1
8	IVIG	6	Yes (stabilization)	–	–	6

*Pt* patient, *IVIG* intravenous immunoglobulin, *AZT* azathioprine.

### SCIg treatment

SCIg treatment was started in all patients a mean of 12.4 years (range 7–22 years) after the disease onset. SCIg were chosen after IVIG because: (1) patients discomfort caused by repeated and long journeys to the infusion site (7/8 pts.), (2) economic burden (5/8), (3) work problems when moving to the infusion site (6/8), (4) difficulties related to venous access (1/8). The mean dose of SCIg used was of 21.7 g/week. Table 3 summarizes the results of the scores applied (ONLS, MRC-SS and LQI variation). However, in 7/8 patients ONLS remained stable or even decreased. We observed an increase of ONLS, representing a sign of clinical deterioration, only in one patient, whereas his result on MRC-SS was equivalent to baseline. Considering MRC-SS, 4/8 patients remained stable and 2/8 patients had a significant increase of 5 and 4 points, consisting with an improvement of their clinical condition, even if ONLS remained unchanged in these two patients. Only two patients presented a slight deterioration (only one point) in MRC-SS. Considering LQI results, a significant increase of patient satisfaction was recorded (mean LQI increase: + 22.5 points). Globally, SCIg length have lasted an average of 6.4 years. Two patients (#1 and 6), during SCIg treatment, experienced some relapse of the disease and needed the administration of IVIG cycle (once every 12–18 months after beginning of SCIg therapy).

**Table 3:** SCIg administration results.

Pt	Dose SCIg (g/week)	Disease duration at T0 (years)	SCIg duration (years)	ONLS		MRC s.s		LQI	
				T0	T1	T0	T1	T0	T1
1	24	17	8	4	3	72	*71*	81	96
2	20	10	6	5	5	69	73	78	93
3	20	8	8	4	4	63	68	62	99
4	30	22	5	5	6	64	64	65	79
5	20	16.5	4.5	3	3	73	72	62	90
6	20	16	7	7	7	33	33	54	81
7	20	2	5	4	4	76	76	70	92
8	20	8	8	4	3	58	58	82	94

*Pt* patient, *ONLS* overall neuropathy limitation scale, *MRC s.s.* medical research council sum score, *LQI* life quality index questionnaire, *T0* baseline (at SCIg treatment beginning), *T1* last follow-up.

### Adverse events

Adverse events (AEs) reported were mostly redness, swelling, induration, and pruritus in the infusion area. We also recorded three serious adverse events (SAEs): pt. 1, 5 years after SCIg starting, experienced a cerebral venous sinus thrombosis, resolved after adequate medical therapy. After this episode, SCIg were suspended for a brief period and restarted once thrombosis disappeared. Pt. 2, 6 years after SCIg start, developed progressive dysphagia, dysphonia and dysarthria. Repetitive stimulation and single fiber electromyography (SFEMG) demonstrated the presence of myasthenia gravis, which caused his death for respiratory failure. Finally, pt. 4 experienced a sudden death of probable cardiac origin five years after SCIg starting.

## Discussion

Few previous studies have showed that SCIg treatment is feasible for MMN. A randomized controlled study was conducted in MMN to compare SCIg and IVIG efficacy on a primary parameter (isokinetic muscle strength) or secondary parameters (MRC score, nerve conduction study, 9-hole-peg test, 10-meterwalking test, P-IgG, anti-GM1). Nine IVIG responsive patients were received SCIg or IVIG at an equivalent dose. Then, after a wash out, they were switched to the other treatment arm. Neither differences in motor performances nor in secondary parameters were reported^13^. This trial was prolonged with an open label extension phase, in which six patients were examined after 3, 6, 12 and 24 months. Muscle strength remained stable but four patients had to increase the dose by 20–25% during the study^10^. In another study, five IVIG-responder patients were switched to SCIg, with an equivalent dose. MRC-SS remained stable in 4/5 during the follow-up of 6 months^14^. In 2011, Misbah and colleagues established a “smooth” protocol of transition from IVIG to SCIg for 8 MMN patients, who started with 25% of the IVIG dose for the first week, then 50% in the second week and later 100% from the third to 24th week. In seven patients who completed the study, the MRC-SS and the disability score did not change during the follow-up, although two patients needed an increase of 25% of the SCIg dose^15^.

In 2014, Cocito et al. studied 21 MMN patients, responsive to IVIG, who were followed up to four months, before being switched to SCIg at a 1:1 dose. At follow-up, ONLS and MRC-SS remained unchanged. An adverse event was registered for one patient, who developed a painful erythema 46 days after being switched to SCIg. Then, he underwent two cycles of IVIG treatment. After that, SCIg was restarted without any more AE^16^. The same Italian cohort was studied for two further years. Primary outcome was the adherence to SCIg therapy. SCIg dose was increased of 15% in one patient, 24 months after having switched therapy. Four patients needed an extra IVIG course between 1 and 12 months after being switched to SCIg. Four patients returned to IVIG treatment because of clinical worsening^17^.

In 2015, Hadden et al. converted 4 MMN patients from IVIG to SCIg at a 1:1 regimen. Motor abilities and disability scores remained unchanged for all the patients. All patients reported a high personal satisfaction for SCIg treatment, which were continued by all of them even after the study period^10^. Finally, in 2015 Katzberg et al. published a series of 15 IVIG responsive MMN patients switched to SCIg with a dose equivalent of 1:1.53. Eleven of the 15 patients completed the six-month study period and remained stable^18^. Considering all these studies, as well as some case reports, it is to be noticed that the longest SCIg treatment follow up lasted no longer than 56 months (4.6 years)^11^.

The eight patients herein reported have been studied for a considerably longer period, lasting 4.5–8 years (mean 6.4). At baseline, they had a long history of MMN with a mean value of 12.4 years (range 7–22 years). Curiously, the three patients who did not undergo IVIG as first line treatment, had the worst ONLS values (≥ 5) at baseline. However, they were also the only three patients presenting comorbidity (diabetes type 2, cutaneous T-cell lymphoma, autoimmune hypothyroidism), that could have influenced their global clinical picture and the response to therapy. Their disease course, in fact, was characterized by an unbalanced disease control, using at least two different drugs or more (cyclophosphamide in three cases; rituximab, azathioprine and interferon-beta in one cases). Even if response to cyclophosphamide was satisfying, with periods of complete disease regression, SAEs led to switch to other therapies. Partial benefit was reported from the patient who underwent rituximab treatment (four cycles); no results obtained with azathioprine or interferon-beta. On the other hand, all patient improved with IVIG treatment that slowed down the disease.

Once converted to SCIg, their responsiveness was quite good. Strength and motor functions remained stable or even improved during this long-term follow-up with benefits on walking capability, resistance to physical efforts and ability in hand fine movements. In fact, in two patients ONLS score reduced of 1 point and other two patients had a significant increase in MRC-SS ≥ 4 points. Only for one patient, many years after SCIg start, we recorded an increase in ONLS, but without variation of MRC-SS result. When this happened, the patient was well over 80 years and surely some concomitant aging-related event contributed to his worsening.

Relapse rate was of 25%: two patients reported periodic disease relapse (every 12–18 months); in these cases, an IVIG course had to be added to obtain clinical improvement. Similarly to what we previously reported about CIDP patients under SCIg treatment^12^, SCIg were usually well tolerated. Mainly, local and rapidly reversible AEs were reported. About the three SAEs reported, two patients (#1, cerebral venous sinus thrombosis; #4, sudden death of probable cardiac origin) could be supposed to be related to the already known pro-thrombotic effects of immunoglobulin therapy, which can cause thrombotic events with an incidence of 1–16.9%^19^. However, the cause of death in patient #1 was not precisely determined, and since he was 85-year-old at the time, his death has been interpreted as an age-related event. Instead, #1 had a documented thrombosis and she needed anticoagulant therapy that is still ongoing.

When requested to compare SCIg and IVIG treatments, the patients reported an increase in global personal satisfaction. They noticed a significant improvement of their quality of life after having switched to SCIg. In particular, they appreciated the possibility of injecting themselves at home, without interruption of working, social or daily life activities and with any necessity to afford extra-costs to reach the infusion site or even to reside nearby for the daily IVIG infusions.

The relatively small number of patients and the retrospective design could be considered as limitations of this study. In addition, the absence of both attempts of withdrawal from SCIg treatment and of a control group, prevent us to definitively declare that SCIg treatment has been fully effective in our patients. A longer follow-up of larger cohorts of MMN patients, in association with attempts either of gradual reduction of SCIg dosage or of cessation of treatment, would be necessary to more precisely evaluate the effects of long-term SCIg treatment.

The main strength of our study is the long-term follow up up to 96 months, during which SCIg therapy has been proved as a safe and tolerable treatment option in MMN patients. Patients’ quality of life clearly improved after switching from IVIG to SCIg that were surely preferred as a chronic treatment. These results strengthen the recommendation to use SCIg as an alternative chronic therapy in patients with MMN previously responders to IVIG courses.

## References

[1] Beadon K, Guimarães-Costa R, Léger JM (2018). Multifocal motor neuropathy. Curr. Opin. Neurol..

[2] Jovanovich E, Karam C (2015). Human immune globulin infusion in the management of multifocal motor neuropathy. Degener. Neurol. Neuromuscul. Dis..

[3] Nobile-Orazio E, Gallia F (2013). Multifocal motor neuropathy: Current therapies and novel strategies. Drugs.

[4] Kumar A, Patwa HS, Nowak RJ (2017). Immunoglobulin therapy in the treatment of multifocal motor neuropathy. J. Neurol. Sci..

[5] Vlam L (2011). Multifocal motor neuropathy: Diagnosis, pathogenesis and treatment strategies. Nat. Rev. Neurol..

[6] Joint Task Force of the EFNS and the PNS (2010). European Federation of Neurological Societies/Peripheral Nerve Society guideline on management of multifocal motor neuropathy. Report of a joint task force of the European Federation of Neurological Societies and the Peripheral Nerve Society-first revision. J. Peripher. Nerv. Syst..

[7] Braine ME, Woodall A (2012). A comparison between intravenous and subcutmaneous immunogobulin. Br. J. Nurs..

[8] Christiansen I, Markvardsen LH, Jakobsen J (2018). Comparisons in fluctuation of muscle strength and function in patients with immune-mediated neuropathy treated with intravenous versus subcutaneous immunoglobulin. Muscle Nerve..

[9] Markvardsen LH, Christiansen I, Jakobsen J (2016). Improvement of hemoglobin levels after a switch from intravenous to subcutaneous administration of immunoglobulin in chronic inflammatory demyelinating polyneuropathy and multifocal motor neuropathy. Transfusion.

[10] Hadden RD, Marreno F (2015). Switch from intravenous to subcutaneous immunoglobulin in CIDP and MMN: Improved tolerability and patient satisfaction. Ther. Adv. Neurol. Disord..

[11] Markvardsen LH, Harbo T (2017). Subcutaneous immunoglobulin treatment in CIDP and MMN. Efficacy, treatment satisfaction and costs. J. Neurol. Sci..

[12] Gentile L, Mazzeo A, Russo M, Arimatea I, Vita G, Toscano A (2020). Long-term treatment with subcutaneous immunoglobulin in patients with chronic inflammatory demyelinating polyradiculoneuropathy: A follow-up period up to 7 years. Sci. Rep..

[13] Harbo T, Andersen H, Hess A, Hansen K, Sindrup SH, Jakobsen J (2009). Subcutaneous versus intravenous immunoglobulin in multifocal motor neuropathy: A randomized, single-blinded cross-over trial. Eur. J. Neurol..

[14] Eftimov F, Vermeulen M, de Haan RJ, van den Berg LH, van Schaik IN (2009). Subcutaneous immunoglobulin therapy for multifocal motor neuropathy. J. Peripher. Nerv. Syst..

[15] Misbah SA (2011). A smooth transition protocol for patients with multifocal motor neuropathy going from intravenous to subcutaneous immunoglobulin therapy: An open-label proof-of-concept study. J. Peripher. Nerv. Syst..

[16] Cocito D (2014). SCIg and chronic dysimmune neuropathies Italian Network. Subcutaneous immunoglobulin in CIDP and MMN: A short-term nationwide study. J. Neurol..

[17] Cocito D (2016). Subcutaneous immunoglobulin in CIDP and MMN: A different long-term clinical response?. J. Neurol. Neurosurg. Psychiatry..

[18] Katzberg, H., Rasutis, V. & Bril, V. Subcutaneous immunoglobulin (IgPRO20) for maintenance treatment in patients with multifocal motor neuropathy (P7.094). *Neurology* **84**(Suppl 14) (2015).

[19] Guo Y, Tian X, Wang X, Xiao Z (2018). Adverse effects of immunoglobulin therapy. Front. Immunol..

